# Winter body mass and over-ocean flocking as components of danger management by Pacific dunlins

**DOI:** 10.1186/1472-6785-10-1

**Published:** 2010-01-21

**Authors:** Ronald C Ydenberg, Dick Dekker, Gary Kaiser, Philippa CF Shepherd, Lesley Evans Ogden, Karen Rickards, David B Lank

**Affiliations:** 1 Centre for Wildlife Ecology, Simon Fraser University, Burnaby, BC V5A 1S6, Canada; 2 3819-112A Street NW, Edmonton, AB T6J 1K4, Canada; 3 402-3255 Glasgow Avenue, Victoria, BC V8X 4S4, Canada; 4 Western and Northern Service Centre, Parks Canada 300 - 300 West Georgia Street, Vancouver, BC V6B 6B4, Canada; 5 Centre for Applied Conservation Research, Forest Sciences Centre, 2424 Main Mall, University of British Columbia, Vancouver, BC V6T 1Z4, Canada

## Abstract

**Background:**

We compared records of the body mass and roosting behavior of Pacific dunlins (*Calidris alpina pacifica*) wintering on the Fraser River estuary in southwest British Columbia between the 1970s and the 1990s. 'Over-ocean flocking' is a relatively safe but energetically-expensive alternative to roosting during the high tide period. Fat stores offer protection against starvation, but are a liability in escape performance, and increase flight costs. Peregrine falcons (*Falco peregrinus*) were scarce on the Fraser River estuary in the 1970s, but their numbers have since recovered, and they prey heavily on dunlins. The increase has altered the balance between predation and starvation risks for dunlins, and thus how dunlins regulate roosting behavior and body mass to manage the danger. We therefore predicted an increase in the frequency of over-ocean flocking as well as a decrease in the amount of fat carried by dunlins over these decades.

**Results:**

Historical observations indicate that over-ocean flocking of dunlins was rare prior to the mid-1990s and became common thereafter. Residual body masses of dunlins were higher in the 1970s, with the greatest difference between the decades coinciding with peak peregrine abundance in October, and shrinking over the course of winter as falcon seasonal abundance declines. Whole-body fat content of dunlins was lower in the 1990s, and accounted for most of the change in body mass.

**Conclusions:**

Pacific dunlins appear to manage danger in a complex manner that involves adjustments both in fat reserves and roosting behavior. We discuss reasons why over-ocean flocking has apparently become more common on the Fraser estuary than at other dunlin wintering sites.

## Background

Starvation and predation risks are both important in the ecology of wintering shorebirds [1-3], and stored body fat appears central to managing these risks. It provides insurance against starvation during periods of food shortage, but increases energetic flight expenditures and reduces predator escape performance [4,5]. Of course the fat reserve must also be built up in the first place, which requires extra foraging and hence predator exposure.

The trade-off theory of fat storage [6,7] holds that the survival-maximizing level is set by its contrasting effects on the probabilities of mortality by starvation and by depredation. Many temperate shorebird species for example, enlarge the fat reserve in mid-winter, increasing protection against the higher incidence of food shortage at that time of year [8]. Decreases in the amount of mid-winter fat over recent decades, documented for both shorebirds [9] and forest birds [10,11], have been linked to the population recovery of raptors since DDT was banned in the 1970s. The interpretation is that increased raptor populations have heightened predation danger, so prey species have lowered the level of winter fat reserves to increase their predator escape ability, at the expense of diminishing their ability to withstand starvation.

Predation danger effects on roosting behavior have also begun to be recognized. Shorebirds are sensitive to the safety features of roost sites, and may travel long distances to utilize safe roosts [12-14]. They sometimes spend part of the high tide period in flight out over open water, rather than roosting on the ground. This has been termed 'roosting on the wing' [15], 'high tide flight' [16], 'airborne roosting' [17], or 'over-ocean flocking' [18]. We use the latter term here. Over-ocean flocking has been described most extensively in British Columbia [[19], see especially [18]], but has also been observed in Britain [15], Washington State [16], California [20], Germany [17] and The Netherlands [21]. Though generally uncommon, over-ocean flocking appears to be a normal part of the behavioral repertoire of some shorebird species. A video sequence can be watched at http://www.sfu.ca/biology/wildberg/species/wesa.html

Most observers [16,17,20] attribute an antipredation function to over-ocean flocking. Prater [15] does not specifically mention predators, but identifies disturbance as the most important factor. Dekker [18] interprets it as a strategy to avoid stealth attacks by falcons and other raptors [22,23] to which shorebirds are vulnerable while roosting or foraging close to shorelines where there is cover from which raptors can attack. Dekker & Ydenberg [24] show that over-ocean flocking increases safety from predators. It can be thought of as an energetically-expensive roosting option, analogous to expending energy to fly to a distant but safer roost site.

In this paper we compile historical information on over-ocean flocking and winter body mass of Pacific dunlins (*Calidris alpina pacifica*) on the Fraser River estuary. At this site, the abundance of peregrine falcons (*Falco peregrinus*) during autumn and winter has risen steadily since the early 1980s [25], and they are important predators of dunlins [24]. We therefore predicted that the winter weight and roosting behavior of Pacific dunlins had changed over this period, shifting the emphasis from starvation avoidance (i.e. 'traditional' ground-based roosting, high winter fat reserves) to predation avoidance (i.e. over-ocean flocking, low winter fat reserves).

## Results

### High tide behavior

We located a total of six studies (see Table 1) that made observations on dunlins on the Fraser estuary prior to 1994. Participants in three of these studies never recorded over-ocean flocking in their notes, or recalled observing it when later interviewed by us. Observers in the three other studies others saw it once, or on occasion. Over-ocean flocking was recorded much more often by later observers. All five of the post-1994 observers report it as a regular or frequent occurrence.

**Table 1:** Studies of Pacific dunlin on the Fraser River estuary made in winter, from 1971 to the present.

Winter	Observer	Methods	Over-ocean flocking?
1971/72	R. Drent	Surveys/observations [42]	No
1976	P. Major	dunlin 3D flock structure [43]	No
1977/80	G. Kaiser	frequent mist-netting [27] (pers. comm.)	seen once
1979/80	K. Fry	regular high tide counts 27 surveys; [19]	seen on occasion during fall migration
1981/84	A. Farr	dunlin foraging ecology [44]	observed when tide and wind very high
1989/90 1990/91	R. Butler	regular high tide counts, 29 surveys [26]	No
1995/98	P. Shepherd	Ph.D. study [31]	See frequently in all years
all years	R. Swanston	many birding visits, plus radar from ferry	first noted March 1996, regular occurrence since (pers. comm.)
all years	R. Butler	many visits	first noted April 26, 1994 (pers. comm.)
1997/2000	L. Evans Ogden	Ph.D. study [39]	seen frequently in all years
1994/2003	D. Dekker	152 observation days [24]	seen regularly in all years
2006 (Jan)	D. Dekker	detailed observations see Fig. 1	Seen 15/17 d, averaging 2.75 h
2005/2007	Y. Zharikov	dunlin feeding dispersion [45]	seen frequently; (pers. comm.)

*'Over-ocean flocking?' *asks whether the observer describes this phenomenon in the report, or, during later interview, recalled seeing it.

Two experienced long-term observers documented their first observations of over-ocean flocking in the mid 1990s. Richard Swanston has for many years made regular birding visits to our study area, and works on the ferry that sails along the study area. He first noted over-ocean flocking by Pacific dunlins in March, 1996 (on the ship's radar) and reports (pers. comm.) that it has since become increasingly common. Dr. Robert Butler (pers. comm.; he also made one of the pre-1994 studies [26]) first observed over-ocean flocking on April 26, 1994. Butler never observed over-ocean flocking in two winters of regular surveys [26], see Table 1] or on many other visits to the Fraser estuary prior to 1994.

**Figure 1:**
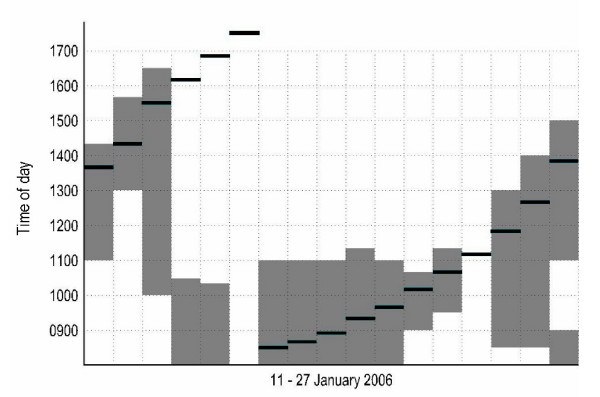
**Over-ocean flocking by Pacific dunlins**. Graphical summary of over-ocean flocking observations by DD made in January, 2006. Columns are successive days, with time given on the y-axis. The horizontal line in each column gives the time of high tide, and the shaded portion indicates the duration of over-ocean flocking. Further details in the text.

During the extended set of observations made by DD in January, 2006, over-ocean flocking was frequent, and prolonged (Fig. 1). It occurred on 15/17 observation days, with the only two days on which it did not occur (January 11 and 24) being, respectively, the only windless day, and the only day with continual heavy rain. The duration of over-ocean flocking ranged from 1.5 - 6.5 h, and the mean duration over all 17 days was 2.8 h, which is similar to Shepherd's (2001a) estimate made (in winter 1995/96, and spring 1998) using radio telemetry of 3.0 h spent in flight each day, the majority during high tide periods. These observations also indicated that over-ocean flocking did not take place at night.

### Winter body mass

**Figure 2:**
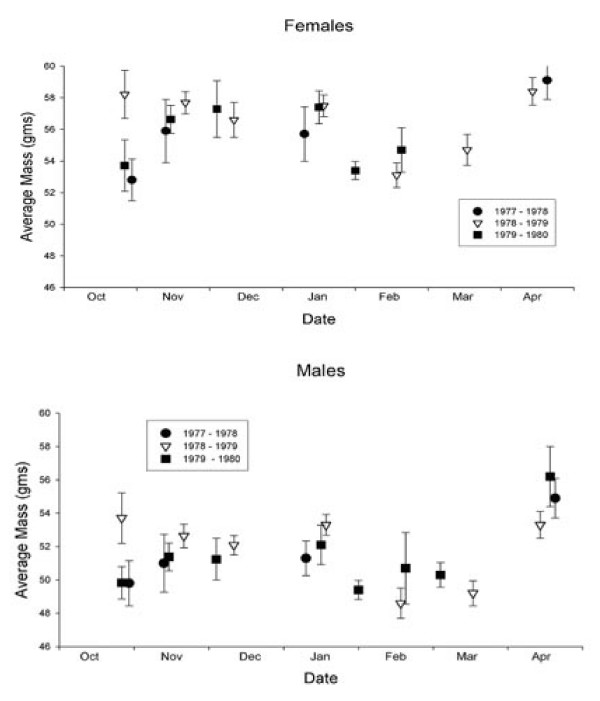
**Winter body mass of Pacific dunlins, by month**. Summary by month of Pacific dunlin mean mass (g) measured on the Fraser River estuary during the winters of 1977/78, 1978/79 and 1979/80, for females (above) and males (below). Overall, the seasonal pattern of weights in the three years is similar. Samples contain only gender-assigned individuals, and thus exclude about 30% of individuals. Exact dates of capture vary somewhat between months and years. Samples sizes for individual entries range from 14 - 130; total sample size = 1883. Error bars are 95% confidence intervals.

As described in the Methods, of the three winters of mist-netting effort in the late 1970s, full data are available only for 1978/79 [see [27]]. Monthly summaries remain for the preceding (1977/78) and following (1979/80) years; these are depicted in Fig. 2 with an analogous monthly summary prepared from data for 1978/79. The seasonal progression of body mass is similar in all three winters, and clearly shows the pattern identified by Kaiser & Gillingham [27]. Body mass is low upon migratory arrival in October, but rises steeply and remains high from November through January. Body mass falls in February and remains low until late in March, when it climbs steeply prior to spring migration. This comparison shows that the 1978/79 data are representative of other years at that time.

**Figure 3:**
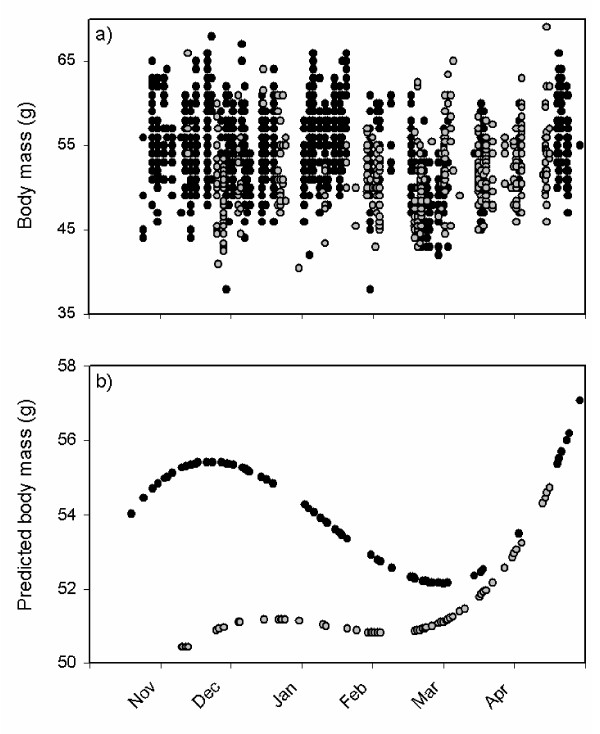
**Winter body mass of Pacific dunlins**. **a) **Masses and **b) **predicted masses generated by third degree b-splines of dunlins captured on the Fraser River Delta during the winters of 1978/79 (in black) and 1994 - 2000 (in grey). See methods for details.

The 1978/79 body mass data are compared with those from the 1990s in Fig. 3a; and decade-specific third-degree splines are shown in Fig. 3b. The spline fitted to the 1970s sample closely matches the pattern described above, but in the 1990s sample the November - December peak in mass has all but disappeared. The body mass difference between the decades is greatest (~4 g) during the autumn, and shrinks over the course of the winter until it disappears in the pre-spring migration period.

Mean culmen and winglengths differ slightly but significantly between decades, though in opposite directions (culmen 0.83 mm [2.15%] shorter in 1990s sample; wing length 1.33 mm [1.09%] longer in 1990s). As expected [28], culmen is a statistical predictor of body mass for both decades (p < 0.0001 for main culmen effect, culmen by decade interaction p = 0.690), but wing length is a significant predictor of body mass only in the 1970s, when birds were relatively heavy (wing by decade interaction, p < 0.0001). To compare the winter masses of Pacific dunlins shown in Fig. 3, we fitted a b-spline to the pooled data. Controlling for culmen and wing length, residual masses are strongly and significantly lower in 1970s than in the 1990s. The overall mean difference is 2.4 g (t = 19.3, df = 1708, p < 0.0001), though as noted above, the difference is biggest in November, and falls to zero or near-zero by late March.

**Figure 4:**
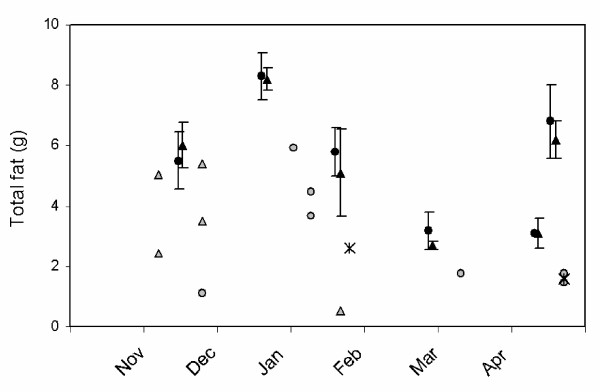
**Whole body fat content of Pacific dunlins**. Distributions of whole body fat content of dunlins collected in the late 1970s (black symbols; from McEwan & Whitehead [29]) versus the 1990s (grey symbols). Error bars are standard deviations. Females are indicated by a circle and males by a triangle. Two individuals from the 1990s dataset could not be gender-assigned and are designated by a star. Details of the data sets are given in the Methods.

Fig. 4 compares the whole-body fat content of 1990s dunlins with that reported by McEwan & Whitehead [29] for the 1970s. The 1990s data are too few for a definitive assessment, but show that all the birds measured in the 1990s carried less fat than the means reported for the 1970s, and that the absolute difference is about 2-4 g, which suggests that the change in body mass between the decades can be accounted for largely by a decline in the amount of fat carried.

### Peregrine seasonal abundance pattern

**Figure 5:**
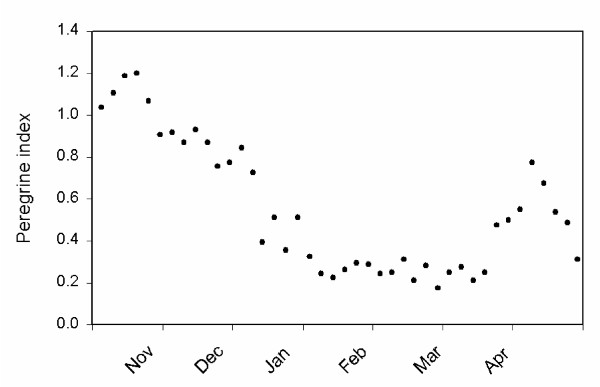
**Seasonal change in peregrine abundance**. Seasonal index of peregrine abundance, based on the mean number of sightings during standardized daily surveys, grouped into successive 5-day periods, 1986 - 2000. The annual peak is reached during October, and declines during the course of the winter. A second peak follows in mid-April. Pacific dunlins' winter residence on the Fraser estuary begins just after the autumn peak, and ends just before the spring peak of the peregrine index. The data were collected by John Ireland, manager of the George C. Reifel Migratory Bird Sanctuary, located on the Fraser estuary. Based on Lank et al. [1].

As documented in Ydenberg et al. [[25]; see Methods], peregrines were uncommon on the Fraser estuary prior to the ban on DDT in 1973. Matching the continental pattern, their numbers began to rise strongly in the late 1970s or early 1980s and have climbed steadily since [25]. Peregrines have a marked seasonal pattern of abundance on the Fraser estuary (Fig. 5), rising steeply during August to the annual peak during October, before declining during the remainder of the winter to an annual low just before spring migration resumes. Peregrines are relatively abundant during the spring migration period (mid-April - mid-May), but few breed locally and they are virtually absent after this time until they begin to reappear in August.

This pattern seems to have emerged as peregrine numbers recovered. Monthly raptor surveys carried out in and around our study area during the winters of 1970-1979 by the Vancouver Natural History Society sighted only 51 peregrines in 416 surveys [30]. The detection rate was steady and low from October to March, indicating that seasonal pattern described by Lank et al. [1] was absent or greatly reduced prior to peregrine population recovery.

## Discussion

The data presented here show that Pacific dunlins wintering on the Fraser estuary in British Columbia have changed aspects of their winter ecology over the past few decades. In the late 1970s they showed classic temperate shorebird winter behavior, with a regular routine of feeding at low tide and ground-based roosting at high tide, and a mid-winter peak in body mass, attributable to fat storage. After the mid-1990s, the mid-winter peak in body mass disappeared, and dunlins began to spend several hours during the high tide period in over-ocean flocking. We interpret these changes as an adaptive response to greatly increased peregrine falcon presence during the winter since the 1970s.

Table 1 summarizes the evidence that over-ocean flocking has become more frequent. For three reasons we feel that we can discount the possibility that over-ocean flocking occurred as frequently during the early (pre 1994) studies, but was missed because early observers were unaware of its occurrence, while later observers had been alerted to this phenomenon. (A) Save Zharikov, none of the later observers was aware of over-ocean flocking when they began their studies (pers. comm.). This is not surprising, as the descriptions of over-ocean flocking available at the time [15,16,19] were not in mainstream publications, and none devotes more than a few lines to the description. (B) None of the 56 sets of systematic high tide surveys documented during the winters of 1979/80 (n = 27; [19]) and 1989/90 and 1990/91 (n = 29; [26]) records a low number of roosting dunlins, as would be expected if the birds were engaged in unseen over-ocean flocking. (C) Two observers with extensive experience predating 1994 (see Table 1) both documented their first observations of over-ocean flocking in the mid-1990s. We thus feel confident in concluding that on the Fraser estuary this phenomenon has greatly increased in frequency, duration, or both, since the late 1970s.

The data presented here also confirm that in the late 1970s dunlins were heavier than in the late 1990s, the difference being greatest (~4 g) in November, and shrinking steadily over the course of the winter until the pre-spring migration period, when masses are the same. This convergence rules out the possibility that the body mass difference is attributable to some systematic bias (e.g. scale calibration). Our measurements of total body fat are consistent with all or most of the body mass difference between the decades being attributable to a decline in fat reserves.

The slight though statistically significant differences in mean culmen length and mean wing length between the decades are likely attributable to differences in details of the measurement (e.g. personnel, instruments, calibration) or sampling (e.g. habitat) procedures (cf. [28]). In a sample of Pacific dunlins collected on the Fraser River estuary in 1992-1995 (i.e. between the samples reported here; see [31]), means for both culmen (2.18%, relative to 1970s sample) and winglength (1.59%) are slightly larger than either sample reported on here. This indicates that the differences between samples do not represent an ongoing change in the size composition of the population, and supports the interpretation that the changes are attributable to minor procedural differences.

The seasonal pattern of mass decline between the decades shows a striking correlation with the seasonal pattern of peregrine occurrence on the Fraser estuary. The biggest mass drop coincides with the autumn peak in peregrine abundance, with the difference shrinking as the peregrine index declines during the winter. Mass in the decades is the same in March, when peregrines are at their annual (near zero) low. This correlation supports our hypothesis that Pacific dunlins have shifted emphasis from protection against starvation to protection against predation. Piersma et al. [9] reached a similar conclusion in their analysis of changes in the mid-winter masses of golden plovers in The Netherlands. As the data are correlational, we must be cautious about inferring causation, or ruling out contributing roles for any of the many other factors that must have changed at this location between these decades.

Another hypothesis to explain the body mass reduction is that starvation risk has declined over recent decades as climate change reduced the severity of winter weather. To evaluate this we obtained weather records for the period 1970 - 2007 from the National Climate Data and Information Archive of Environment Canada. In Table 2 we summarize the rate of change in average daily temperature, total precipitation, and maximum wind gust on the study area over the winter months. These data provide weak support at best for the idea that winter weather has become less severe. Though the change in mean daily temperature is slightly positive for each winter month over this period, ranging from 0.01 to 0.07°C y^-1^, neither the change in total monthly precipitation nor gustiness are consistent in direction across months, and the low r^2 ^values in Table 2 indicate that all three metrics are very noisy. In fact, the total net change over the 37 year record is much smaller than most of the year to year changes recorded.

**Table 2:** Climate change during winter on the Fraser estuary

Month	Daily mean temperature (°C)			Total precipitation (mm)		
	Mean	Slope	r^2^	Mean	Slope	r^2^
November	6.07	0.03	0.06	180	0.97	0.02
December	3.71	0.04	0.07	174	-0.80	0.02
January	3.59	0.07	0.18	160	1.89	0.12
February	4.78	0.01	0.01	112	-1.54	0.10
March	6.64	0.03	0.09	114	0.33	0.01
**Month**	**Maximum wind gust (km h^-1^)**					
	**Mean**	**Slope**	**r**^**2**^			
November	67.9	0.16	0.02			
December	69.5	0.21	0.02			
January	67.6	0.20	0.03			
February	62.6	-0.11	0.01			
March	68.5	-0.11	0.01			

Summary by month of daily mean temperature, total precipitation, and maximum wind gust speed, during 1970 - 2007, from the National Climate Data and Information Archive of Environment Canada http://www.climate.weatheroffice.ec.gc.ca/, recorded at the Vancouver International Airport, located adjacent to the Fraser estuary. The table records for each winter month the overall mean, the slope of the regression on year (1970 to 2007), and the proportion of the variance explained (r^2^).

Neither does the seasonal pattern of change in body mass between the decades match very closely the pattern in those climate measures that do show evidence of change. The biggest seasonal mass change between the 1970s and 1990s occurs in the autumn, with the difference shrinking until March, when masses are the same. In contrast January shows the strongest rate of increase in temperature since 1970, followed by March. Changes in precipitation and wind match even less well. Overall, we feel that the reduction in winter body mass of Pacific dunlins is not well-explained by an hypothesis based on reduced winter severity. Neither is this hypothesis able to explain why over-ocean flocking has become commonplace.

We regard the changes in roosting behavior and body mass as adaptive adjustments to increased danger. Other interpretations are that the body mass decline is a non-strategic consequence of extra flight induced by harassment from numerous peregrines, or that it is a strategic reduction of body mass made to reduce flight costs because flight time has increased for a reason unrelated to predation danger. Differentiating between these competing hypotheses could be undertaken with an analysis of when strategic over-ocean flocking (or more generally, roosting behavior) ought to occur. The first study to make a strategic analysis of roost site choice is that by Rogers et al. [13]. In their study area, distant roost sites required more travel than nearby roost sites, but birds suffered fewer disturbances there. They accounted for roost choice with a model that minimized total energy expenditure over the entire high water period, summing the energetic costs of both travel to the roost site and time spent in flight while there. We agree with this general approach, but feel that the choice is more appropriately analyzed in terms of maximizing survival than in minimizing energy expenditure. Over-ocean flocking becomes worthwhile when it reduces the probability of mortality [20], taking account of the extra mortality that results from the extra foraging required.

Several factors may dispose dunlins on the Fraser estuary to more prolonged and frequent over-ocean flocking than at other sites. First, the winter population of peregrines is high relative to other locales, and the danger they pose to dunlins wintering there is particularly great, because kleptoparasitic competition from Bald Eagles (*Haliaeetus leucocephalus*) forces peregrines to concentrate their foraging on dunlins instead of ducks, a favorite prey for peregrines elsewhere [32-34]. Second, the nature of the Fraser River estuary is such that alternative coastal and inland roosting sites are also dangerous, due to the presence of peregrines, merlins (*Falco columbarius*) and northern harriers (*Circus cyaneus*), all of which prey on dunlins [18]. Finally, the Fraser estuary is large and highly productive, and the winter in southwest British Columbia generally mild, which makes it possible to finance the extra energetic expenditure required. Dunlins cease over-ocean flocking during extended periods of freezing weather [18], on days with heavy rain (see Fig. 1) and on windless days, which suggests that increases in the energy requirement (freezing weather, rain) or the cost of the gliding and hovering mode of OOF flight (lack of wind) make it too expensive. Analyzing over-ocean flocking on the Fraser estuary and on other wintering areas in this way should lead to further understanding of when and why it is observed.

## Conclusions

Pacific dunlins (*Calidris alpina pacifica*) wintering on the Fraser River estuary in southwest British Columbia altered aspects of their wintering ecology between the 1970s and 1990s. We found that Pacific dunlins were lighter by several grams in the 1990s as compared with the 1970s. The difference appears largely or entirely attributable to a reduction in fat reserves. Historical observations indicate that over-ocean flocking in place of roosting at high tide was rare prior to the mid-1990s and became common thereafter. We interpret these changes as a shift by dunlins toward increased protection against predation, due to the increase since the 1970s in the abundance of Peregrine falcons (*Falco peregrinus*). Fat stores offer protection against starvation, but are a liability in escape performance, and increase flight costs. 'Over-ocean' or 'high tide' flocking is a relatively safe but energetically-expensive alternative to roosting during the high tide period. Shifting use of these tactics alters the balance of protection against predation vs. starvation risks.

## Methods

### General

Pacific dunlins winter at sites along the Pacific coast from southwest British Columbia to Baja California. Some 20-40,000 spend the winter period (November - March) on the Fraser River estuary in southwest British Columbia. As many as 100,000 or more may be present in October during southward migration from Alaskan breeding grounds [35,36], and in April during the return northward migration. The tidal regime is semi-diurnal and winter low tides occur mostly at night. At high tide, dunlins roost along the foreshore, or on very high tides in the adjacent saltmarsh and nearby fields. Roosting flocks may form almost anywhere on the nearly-continuous 25 km of foreshore along the estuary edge. The observations and counts of dunlins reported in the various studies cited below were made from the dyke that borders the estuary.

### Over-Ocean Flocking

Flocks of shorebirds are well-known to respond to raptor attacks with spectacular flight maneuvers. The flight mode during over-ocean flocking is very different. The earliest description we could find is in an unpublished report by Fry [19] on the wintering ecology of Pacific dunlins on the Fraser estuary. She writes that ' [over-ocean flocking] *was.... noted during *[autumn] *migration when flocks did not roost at high tide - instead they formed extensive, widespread hovering clouds of birds high over the bay, occasionally roosting for short periods but displaying restlessness*.' Dekker's [18] description, made at the same site, says '....*the majority of the dunlins flew out over the ocean, where they coursed back and forth. The flock drifted in a loose cloud on the wind or coalesced into a dense undulating stream low over the waves*...'. These attributes can be clearly observed on the video sequence at http://www.sfu.ca/biology/wildberg/overoceanflocking.html

Over-ocean flocking is also prolonged relative to predator escape flights. Hötker [17] distinguished 'short flights caused by an actual attack of a raptor' from 'airborne roosting' by the length of flight (at least 30 min). Over-ocean flocking can be difficult to spot because birds fly far out over the ocean, may fly very high or very low, and the flight involves 'hovering' or 'drifting' rather than the conspicuous, flashing turns of shorebird flocks evading predators. However, it may last throughout the high tide period (see below), and thus affords ample opportunity to be observed if it does occur.

We located reports from all the field studies and observations of wintering Pacific dunlins on the Fraser River estuary made since 1970, documented in published as well as unpublished sources. All these studies involved experienced observers making frequent and regular visits to the foreshore during high tide periods to census numbers, to mist-net birds for banding or to collect other data, all of which created opportunities to see over-ocean flocking if it did occur. We interviewed all the investigators we could trace about their recollections of this behavior, and also contacted other knowledgeable observers who had made frequent visits to the estuary. These studies and contacts are summarized in Table 1.

Detailed observations of over-ocean flocking were carried out by one of us (DD) in the course of an observational study of the hunting habits of peregrines. In eight winters from 1994 to 2006, DD made 169 high-tide visits to the foreshore of the Fraser estuary, logging more than 1000 observation hours [see [18,24,34]]. During the 17 sequential days of observation (January 11-27, 2006) reported here, he monitored dunlin flocks from a vantage point on the dyke. The onset and cessation of over-ocean flocking were recorded.

### Winter Mass

Pacific dunlins were captured on the Fraser estuary during the winters of 1977/78, 1978/79 and 1979/80 by GK (n = 2680; see [27]). Captures were made using mistnets on mudflats at diurnal high tides. Each bird was weighed (± 1.0 g), and the exposed culmen (± 0.1 mm) and wing (± 1.0 mm) were measured. Sex was assigned based on culmen length (≤ 37.7 mm = male; 37.8 mm - 39.7 mm = unassigned; ≥ 39.8 mm = female) following Page [37]. Note that by this method almost 30% individuals can not be sex-assigned. Complete data for 1316 individuals captured in winter 1978/1979 are in the archives of the Canadian Wildlife Service. Unfortunately, the original data for individuals captured in the preceding (1977/78) and subsequent winter (1979/80) could not be located, though a summary table of mean monthly weights remains ([27]; Figs 1 and 2). We compare this to an analogous summary prepared from the detailed data for winter 1978/79, to help establish whether the latter year was perhaps unusual.

Pacific dunlins were again captured on the Fraser estuary during the winters of 1994/95, 1995/96 and 1997/98 for a radio-tracking study by PS ([31,38]; n = 111), and during the winters of 1997/98, 1998/99 and 1999/2000 for a feeding study by LEO ([39,40]; n = 295). Capture methods differed from the 1970s in that birds were mist-netted not only on mudflats, but in adjacent fields on nocturnal high tides. Birds were weighed and measured following the same protocol as in the 1970s (i.e. following [37]; see [28]). We compare morphometrics of dunlins from the 1970s (GK's 1978/79 sample) with those from the 1990s (birds caught by PS and by LEO). Some measures are missing for a few individuals, so sample sizes vary slightly in some of the comparisons made below.

We summarized seasonal patterns of body mass by generating date-specific predicted values using b-spline procedures implemented by PROC TRANSREG in SAS^® ^[41], generating a third degree equation with no knots to allow for two inflection points over the winter. We calculated separate splines for each decade to compare patterns visually.

To test for statistical differences between decades, we first controlled for variation in body size by regressing mass against culmen length, winglength, and decade. Predicted mass values from this regression were used as input for values of a seasonal spline calculated on data pooled over decades. We then tested for decade effects on date-specific residuals from this spline.

McEwan & Whitehead [29] analyzed the whole-carcass body composition (water content, fat-free dry mass, fat content) of 171 dunlins shot on the Fraser River estuary on six dates in the winter of 1979/80. (These birds form a separate sample from that reported in Kaiser & Gillingham [27].) We measured the amount of fat carried by dunlins in the 1990s from birds collected in the field by PS and LEO. All were either mist-net mortalities, or found as fresh carcasses under a set of high-tension electrical lines along one of the jetties which dunlin flocks routinely crossed. We presumed these birds were killed when they struck the wires in flight. Parts of many of these 64 carcasses were used in other studies, but the whole-body fat content could be measured from a sample of 14. Following the method described by McEwan & Whitehead [29] permits a direct comparison.

### Peregrine Abundance

Since 1986, near-daily surveys for peregrines have been made by manager John Ireland at the George C. Reifel Migratory Bird Sanctuary, located on the Fraser estuary. In accord with the continental recovery of peregrine populations, the number of peregrines sighted has risen steadily. Also in accord with the continental pattern, the increase began in the late 1970s or early 1980s [25], from a near-zero level. Peregrine abundance during the winter and adjacent migration periods (October through April) was indexed by the daily number of peregrines sighted, calculated over 5 d periods, and averaged over all years (1986 - 2000). The data used here are presented in Figure three of Lank et al. [1].

## List of Abbreviations

OOF: over-ocean flocking; defined in Methods.

## Authors' contributions

RCY conceived and co-ordinated the study, assembled the diverse data sets, located and interviewed the authors of previous studies, and wrote the manuscript. DD carried out almost all the direct observation of OOF, including the data reported in Fig. 1. GK, PCFS and LEO collected and contributed the data on Pacific dunlin body mass. KR carried out the analyses of whole body fat. DBL managed all of the data, designed and performed the statistical analyses, and helped to draft the manuscript. All authors read and approved the final manuscript.
